# Supplementary material for: Using eDNA techniques to find the endangered big-headed turtle (Platysternon megacephalum)
Source: PLoS One. 2022 Feb 7;17(2):e0262015. doi: 10.1371/journal.pone.0262015 (PMC8820637; doi:10.1371/journal.pone.0262015)
Supplement: Attachment — Submitted filename: Response to Reviewers.docx [file pone.0262015.s005.docx]

**Reviewer #1: General comments

I have reviewed the manuscript ‘Using eDNA techniques to find the endangered big-headed turtles (Platysternon megacephalum)’. I am largely satisfied with the changes the authors have made to the manuscript, but still have a number of minor comments I would like the authors to address to help improve the clarity of the manuscript. I have detailed these in the specific comments to the authors below. I also recommend that the authors complete the validation checklist provided in Thalinger et al. (2021) for the assay used and provide this as a supplementary file.

Thalinger, B., Deiner, K., Harper, L.R., Rees, H.C., Blackman, R.C., Sint, D., Traugott, M., Goldberg, C.S. & Bruce, K. (2021) A validation scale to determine the readiness of environmental DNA assays for routine species monitoring. Environmental DNA, 3, 823–836.**
[**https://doi.org/10.1002/edn3.189**](https://doi.org/10.1002/edn3.189)

RESPONSE:
Revised as suggested.

We appreciated the recommendation from the reviewer, and completed the validation checklist provided by the literature suggested. The checklist is attached as supplementary table 1.

**Specific comments

Line 19: Change ‘survey’ to ‘surveyed’.**

RESPONSE:
Revised as suggested.

**Lines 22-23: Change ‘eDNA quantity and the environmental variables tested had no significant 23 relationship’ to ‘****There was no significant relationship between eDNA quantity and the environmental variables tested.**
RESPONSE:
Revised as suggested.

**Line 26: Remove comma.**
RESPONSE:
Revised as suggested.

**Line 81: Remove comma before ‘using’**
RESPONSE:
Revised as suggested.

**Line 89: Insert ‘to’ after ‘Prior’.**

RESPONSE:
Revised as suggested.

**How long was equipment immersed in 10% bleach solution for sterilisation?**

We immersed equipment in 10% bleach solution for at least 5 minutes for sterilization each time. We clarified the details in the corresponding sentences.

**Line 96: Insert ‘the’ before ‘fluorometer’.**
RESPONSE:
Revised as suggested.

**Line 99: Change ‘the process’ to ‘sample processing’.**
RESPONSE:
Revised as suggested.

**Line 100: Insert ‘23’ before ‘extractions’.**
RESPONSE:
Revised as suggested.

**Line 107: Remove ‘a’ before ‘reliable’.**
RESPONSE:
Revised as suggested.

**Lines 108-111: I appreciate that the authors will not be repeating qPCR at this stage due to cost and time, but one way of increasing assay sensitivity would have been to use larger reaction volumes for qPCR.**
RESPONSE:
Thank you for your suggestion, we will keep this in mind for the future.

**Lines 111-112: What volume was used for the** **TaqMan Exogenous Internal Positive Control assay in qPCR reactions?**
RESPONSE:

TaqMan Exogenous Internal Positive Control assay composed of two parts: IPC-Mix and IPC-DNA. Following manufacturer’s instruction, we added 1 μL of IPC-Mix and 0.2 μL of IPC-DNA into each reaction, We clarified the details in the corresponding sentences.

**Line 128: Remove comma after ‘uncertain’.**
RESPONSE:
Revised as suggested.

**Line 129: Change ‘of’ to ‘performed on’.**

RESPONSE:
Revised as suggested.

**Line 147: Change ‘eDNA water samples’ to ‘****water samples for eDNA analysis’. Insert ‘walked’ before ‘along’.**

RESPONSE:
Revised as suggested.

**Line 153: Insert ‘(‘ before ‘1)’.**
RESPONSE:
Revised as suggested.

**Lines 181-183: I suggest joining these sentences with the previous paragraph.**
RESPONSE:
Revised as suggested.

**Lines 191-190: Change ‘all negative controls exhibited no qPCR fluorescence signal’ to ‘****none of the negative controls exhibited qPCR fluorescence signal’.**

RESPONSE:
Revised as suggested.

**There is a slight issue here as the qPCR standard curve used in the present study only went down to 100 copies/ul. This is the limit of quantification in this study as 100 copies/ul was the last point on the standard curve against which eDNA samples were being compared. If samples quantified below this, you can only somewhat rely on the copy number estimates produced for these samples as they are outwith the range of the standard curve. It is not good practice to use a standard curve from other studies (e.g. Lam et al. 2020) as it does not accurately reflect the qPCR reactions performed in the present study. I suggest the authors clarify whether their samples were above or below 100 copies/ul. If below, they can still count these as positive detections as there is clearly eDNA present, but these samples shouldn’t really be used in analyses examining eDNA quantity.**

RESPONSE:
We follow the suggestion from the reviewer, indicating whether the samples were above the limit of quantification (100 copies/μL) in text, and in Figure 2. We also remove samples with below 100 copies/μL from statistical analysis. Upon the removal of the data, eDNA quantity and turtle capture rate had no significant relationship anymore (*r_s_* = 0.267, *p* = 0.056). We updated the latest result in the Result section.

**Line 243: Change ‘, but, a significant, positive’ to ‘, but a significant positive’.**
RESPONSE:
Revised as suggested.

**Line 246: Change ‘in’ to ‘on’.**
RESPONSE:
Revised as suggested.

**Line 248: Change ‘them was statistically significant in affecting’ to ‘****these covariates significantly influenced’.**
RESPONSE:
Revised as suggested.

**Line 276: Change ‘species specific’ to ‘species-specific’.**
RESPONSE:
Revised as suggested.

**Line 293: Change ‘Degradation’ to ‘degradation’.**
RESPONSE:
Revised as suggested.

**Line 298 and 308: Change ‘Production’ to ‘production’.**
RESPONSE:
Revised as suggested.

**Line 310: Change ‘increases’ to ‘increase’.**
RESPONSE:
Revised as suggested.

**Line 313: Change ‘produce less eDNA by shedding’ to ‘shed less eDNA’.**
RESPONSE:
Revised as suggested.

**Line 323: Change ‘in hopes’ to ‘in the hope’.**
RESPONSE:
Revised as suggested.

**Line 326: Change ‘Transport’ to ‘transport’.**
RESPONSE:
Revised as suggested.

**Line 328: Change ‘which’ to ‘where’.**

RESPONSE:
Revised as suggested.

**Line 329: Change ‘cause eDNA distribution vary’ to ‘can cause eDNA distribution to’.**
RESPONSE:
Revised as suggested.

**Line 340: Change ‘Dilution’ to ‘dilution’.**
RESPONSE:
Revised as suggested.

**Line 345: Change ‘amplifying’ to ‘resuspending’.**
RESPONSE:
Revised as suggested.

**Line 347: Change ‘model’ to ‘models’.**
RESPONSE:
Revised as suggested.

**Line 363: Change ‘result’ to ‘results’.**
RESPONSE:
Revised as suggested.

**Line 368: Change ‘volume’ to ‘volumes’.**
RESPONSE:
Revised as suggested.

**Line 375: Insert ‘of’ after ‘detection’.**
RESPONSE:
Revised as suggested.

**Line 376: Change ‘present/absent’ to ‘presence/absence’.**
RESPONSE:
Revised as suggested.

**Line 389: Insert ‘reaction volume’ before ‘and’.**

RESPONSE:
Revised as suggested.

**Reviewer #2: The authors provided thoughtful and thorough revision of their paper. Great work!**

**However, I still have some confusion around reporting of assay performance and present minor comments below.

L89: Add “to” to this sentence: “Prior to each lab work step,..”**
RESPONSE:
Revised as suggested.

**L147-148: Suggest revising sentence to read something like, “For active searching, we looked for P. megacephalum along the stream at night using headlamps.”**
RESPONSE:
Revised as suggested.

**L149: Change “was” to “were”**
RESPONSE:
Revised as suggested.

**L173: Delete extra “.” after “...and all KP streams. (n=8).”**
RESPONSE:
Revised as suggested.

**Reporting of qPCR metrics (R2, %efficiency and LOD):
1. Because the authors quantify, both the limit of detection and limit of quantification need to be reported. So the limit of quantification also needs to be reported. Additionally, the copy number estimates need to be reported as reference to the LOD in the paper cited copies/μL.**
RESPONSE:
We apologize for the confusion between LOD and LOQ. In this study, we used 100 copies/μL as the lowest point of standard curve, hence, the limit of quantification. In Lam et al. (2020), the limit of detection was tested to be 10 copies/μL. We clarified the limit of detection in the Methodology section – Quantitative PCR, and the limit of quantification in the Methodology section - Exploration of eDNA Data.

**2. I am confused about the multiple R2 and % efficiency reported, at L106 & L385 (R2=0.97, efficiency=94.15%), L189-190 and Supplemental Table 1 (R2=0.957 ± 0.03; efficiency: 96.99% ± 5.19), and the discussion of lower values at L386. I think reporting of the values only from the qPCR runs in this study should be reported in the results section and then mention the comparison with the previous study in the limitation sections (L381-390).**
RESPONSE:
We apologize for making the confusion. We removed qPCR values (R2 and % efficiency) from other studies from the methodology and result section to minimize confusion. All comparison of qPCR values can be only found in Discussion – Limitation of the study sections as suggested.

**3. As a reader, I am also confused about the reported LOD at 10 copies/μL at L191. In the paper and in the authors’ response it looks like the limit of detection run in this study was 100 copies/μL (L116). I am not sure that LOD is necessarily transferable from study to study, especially given that the R2 and % efficiency for some runs were lower than what would be recommended to quantify eDNA.**
RESPONSE:
To be conservative, we removed amplified eDNA samples with quantity below 100 copies/μL from the analysis. Upon the removal of the data, eDNA quantity and turtle capture rate had no significant relationship anymore (*r_s_* = 0.267, *p* = 0.056). We updated the latest result in the Result section.

**Results:
L238-240: Add the rs and p values here for the correlation relationships between temperature and pH.**
RESPONSE:
The corresponding *r_s_* and *p* values were added into the sentences suggested.

**I had previously suggested that occupancy modeling might be useful in determining which factors are important to detect turtle eDNA. I sincerely apologize if this was not clear. But there is not enough explanation of the parameters included and the results are not fully explained. For example, use of AICc and p-values are generally not used together, there is no null model or global model included. As these relationships are still compared with correlations at L238-240, I suggest deleting this section (246-249), as it's not completely explained.**
RESPONSE:
We sincerely apologize for not enough explanation on the parameters. However, as the reviewer said, the relationship was compared with correlations at L238-240, we followed the suggestion from the reviewer, to delete the section, and the corresponding paragraph in the Methodology section.
