# Supplementary material for: Using eDNA techniques to find the endangered big-headed turtle (Platysternon megacephalum)
Source: PLoS One. 2022 Feb 7;17(2):e0262015. doi: 10.1371/journal.pone.0262015 (PMC8820637; doi:10.1371/journal.pone.0262015)
Supplement: Attachment — Submitted filename: Response to Reviewers.docx [file pone.0262015.s007.docx]

**Reviewer #1: General comments

I have reviewed the manuscript ‘Using eDNA techniques to find the endangered big-headed turtles (Platysternon megacephalum)’. I am largely satisfied with the changes the authors have made to the manuscript, and recommend the manuscript be published after minor comments are addressed. I have detailed these in the specific comments to the authors below.**

**Specific comments

Line 99: Add number of sampled in a set for DNA quantification, i.e. ‘one for each set of X samples for DNA quantification’.**
RESPONSE:
Revised as suggested.

**Line 100: Change ‘2-3’ to ‘two or three’.**
RESPONSE:
Revised as suggested.

**Line 107: Change ‘qPCR assay provided’ to ‘the selected qPCR assay’. Change ‘supplementary table’ to ‘Supplementary Table’.**
RESPONSE:
Revised as suggested.

**Line 113: Change ‘2-3’ to ‘two or three’.**
RESPONSE:
Revised as suggested.

**Line 114: Change ‘are’ to ‘were’.**
RESPONSE:
Revised as suggested.

**Line 124: Change ‘2-5’ to ‘two to five’.**
RESPONSE:
Revised as suggested.

**Lines 125-126: Change ‘when the qPCR assay recovered DNA amplification’ to ‘when qPCR amplification was observed’.**
RESPONSE:
Revised as suggested.

**Line 127: Change ‘is’ to ‘was’.**
RESPONSE:
Revised as suggested.

**Line 129: Change ‘performed on all qPCR reactions of the sample’ to ‘of all qPCR replicates performed on the sample’.**
RESPONSE:
Revised as suggested.

**Line 133-134: Remove ‘were’. Change ‘had zero amplification’ to ‘did not amplify’.**
RESPONSE:
Revised as suggested.

**Line 135: Change ‘2-3’ to ‘two or three’.**
RESPONSE:
Revised as suggested.

**Lines 136 and 137: Change ‘showed amplification’ to ‘amplified.**
RESPONSE:
Revised as suggested.

**Line 144: Change ‘1-2’ to ‘one to two’.**
RESPONSE:
Revised as suggested.

**Line 155: Change ‘Shapiro-Wilk’s’ to ‘Shapiro-Wilk’.**
RESPONSE:
Revised as suggested.

**Line 162: Change ‘quantity data’ to ‘concentration’.**
RESPONSE:
Revised as suggested.

**Line 203: Change ‘2-5’ to ‘two to five’.**
RESPONSE:
Revised as suggested.

**Line 220: Change ‘3 HR, 5 UN’ to ‘three HR, five UN’.**
RESPONSE:
Revised as suggested.

**Line 223: Change ‘6 HR’ to ‘six HR’.**
RESPONSE:
Revised as suggested.

**Line 237: Change ‘[‘ to ‘(‘.**
RESPONSE:
Revised as suggested.

**Line 238: Remove ‘)’. Insert ‘:’ after ‘streams’.**
RESPONSE:
Revised as suggested.

**Line 239: Change ‘]’ to ‘)’.**
RESPONSE:
Revised as suggested.

**Line 256: Change ‘1-2’ to ‘one to two’.**
RESPONSE:
Revised as suggested.

**Line 280: Change ‘in’ to ‘with’.**
RESPONSE:
Revised as suggested.

**Line 281: Change ‘organism’ to ‘individuals’.**
RESPONSE:
Revised as suggested.

**Line 360: Change ‘1-2L’ to ‘1-2 L’.**
RESPONSE:
Revised as suggested.

**Line 378: Change ‘suggest’ to ‘suggests’.**
RESPONSE:
Revised as suggested.

**Line 647: Change ‘of qPCR assay’ to ‘for the qPCR assay’.**
RESPONSE:
Revised as suggested. **Supplementary Table 1: The assay validation checklist from Thalinger et al. (2021) has not been completed properly. Please complete the ‘Checklist template’ sheet in the Excel spreadsheet titled ‘Appendix S1’ that was uploaded as part of the Supporting Information for Thalinger et al. (2021).

Thalinger, B., Deiner, K., Harper, L.R., Rees, H.C., Blackman, R.C., Sint, D., Traugott, M., Goldberg, C.S. & Bruce, K. (2021) A validation scale to determine the readiness of environmental DNA assays for routine species monitoring. Environmental DNA, 3, 823–836.**
[**https://doi.org/10.1002/edn3.189**](https://doi.org/10.1002/edn3.189)

RESPONSE:
Revised as suggested. An updated checklist as Supplementary Table 1 will be submitted with the manuscript.

**Reviewer #2: (No Response)**
